# Research progress on the improvement of cardiovascular diseases through the autonomic nervous system regulation of the NLRP3 inflammasome pathway

**DOI:** 10.3389/fcvm.2024.1369343

**Published:** 2024-04-08

**Authors:** Yuchi Hu, Songyuan Dai, Lulu Zhao, Ling Zhao

**Affiliations:** Department of Cardiology, First Affiliated Hospital of Kunming Medical University, Kunming, Yunnan, China

**Keywords:** NLRP3, ANS, VNS, SNS, cardiovascular disease

## Abstract

Cardiovascular disease stands as a leading global cause of mortality. Nucleotide-binding Oligomerization Domain-like Receptor Protein 3 (NLRP3) inflammasome is widely acknowledged as pivotal factor in specific cardiovascular disease progression, such as myocardial infarction, heart failure. Recent investigations underscore a close interconnection between autonomic nervous system (ANS) dysfunction and cardiac inflammation. It has been substantiated that sympathetic nervous system activation and vagus nerve stimulation (VNS) assumes critical roles withinNLRP3 inflammasome pathway regulation, thereby contributing to the amelioration of cardiac injury and enhancement of prognosis in heart diseases. This article reviews the nexus between NLRP3 inflammasome and cardiovascular disorders, elucidating the modulatory functions of the sympathetic and vagus nerves within the ANS with regard to NLRP3 inflammasome. Furthermore, it delves into the potential therapeutic utility of NLRP3 inflammasome to be targeted by VNS. This review serves as a valuable reference for further exploration into the potential mechanisms underlying VNS in the modulation of NLRP3 inflammasome.

## 1 Introduction

Cardiovascular diseases exhibit high global prevalence (57.2%) (1), with escalating incidence and mortality rates (2). Extensive research has clarified the pathological mechanisms at the core of these diseases, encompassing myocardial cell apoptosis, ventricular remodeling, myocardial inflammatory infiltration, fibrosis, and metabolic disorders (3–6). Notably, Inflammation can induce damage to cardiomyocytes, leading to cardiac dysfunction and disease progression (7). It is crucial to recognize that inflammatory infiltration in myocardial tissue can also contribute to myocardial fibrosis, myocardial cell apoptosis, and disruptions in energy metabolism (8). This inflammatory process stands as a pivotal factor contributing to an unfavorable prognosis and elevated mortality rates in cardiovascular diseases. Mounting evidence emphasizes the significance of the inflammatory cascade reaction initiated by NLRP3 inflammasome activation, serving as a major driver of inflammatory infiltration within myocardial tissue, and a key contributor to specific cardiovascular disorder progression, such as myocardial infarction, heart failure (9–11). Consequently, intervening in the pathological progression in cardiovascular disorders by modulating NLRP3 inflammasome pathway emerges as a crucial protective measure.

Currently, ANS served as pivotal avenue for cardiovascular diseases treatment (12). Numerous studies, particularly in areas including myocardial infarction (MI), as well as heart failure, have illuminated the crucial role of interfering with the NLRP3 pathway in mediating therapeutic effects through the ANS (13). This article aims to comprehensively review the intricate correlation between cardiovascular disorders and NLRP3 inflammasome, delving into the regulatory functions exerted by sympathetic and vagus nerves within the ANS on NLRP3 inflammasome. Furthermore, it explores potential therapeutic targets for VNS in modulating NLRP3 inflammasome, offering valuable insights for subsequent interventions in cardiovascular diseases through ANS-mediated NLRP3 inflammasome pathways.

## 2 NLRP3 inflammasome: new-established therapeutic target within cardiovascular diseases

### 2.1 NLRP3 inflammasome

**Figure 1:**
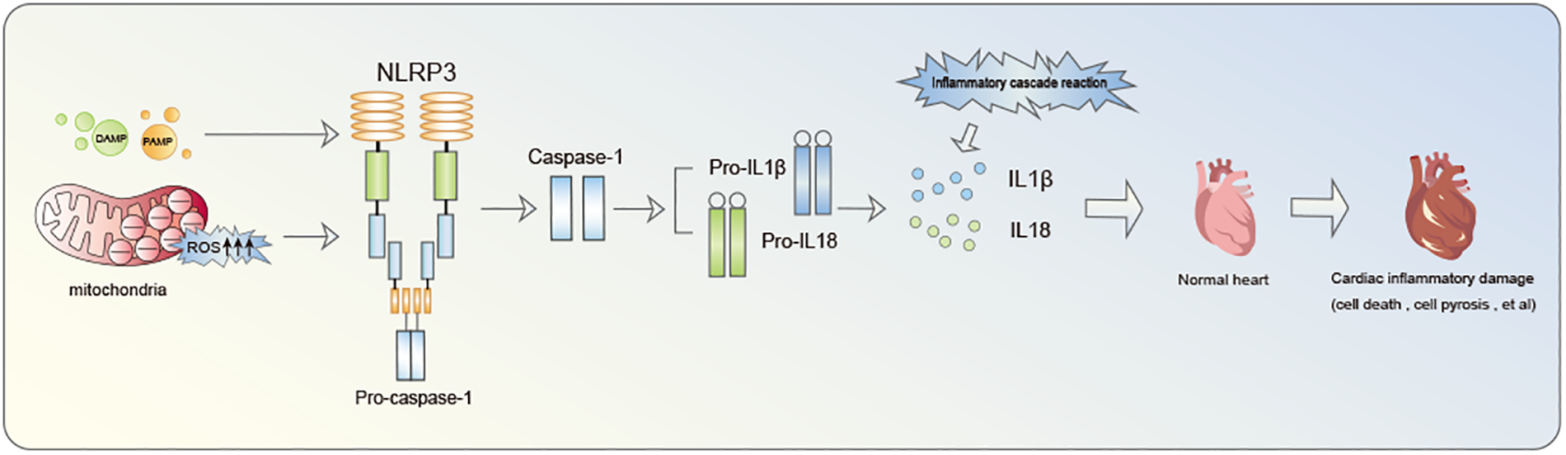
The inflammatory cascade initiated by NLRP3 inflammasome notably contributes to cardiac damage.

Inflammasome is a multiprotein oligomer present in immune cells comprising Nucleotide-binding oligomerization domain (NOD)-like receptors (NLR), Pyrin domain (PYD), and caspase activation and recruitment domain (CARD). NLRP3 inflammasome constitutes a focal point of research in cardiovascular diseases (14). Notably, extensive investigation have been conducted on NLRP3 inflammasome. NLRP3, identified as a nod-like receptor protein, functions as an intracellular pattern recognition receptor primarily linked to the inflammasome pathway. It possesses the ability to detect both pathogen-associated molecular patterns (PAMP) and damage-associated molecular patterns (DAMP) (15, 16). The primary defensive response during infections or tissue damage entails the discharge of cellular contents into the myocardial interstitium and the initiation of intracellular stress pathways. These pathways encompass Reactive oxygen species (ROS) production, oxidative stress, autophagy, and innate immunity triggering. Collectively, these processes contribute to the triggering of NLRP3 inflammasome (17–19). Mitochondrial fission plays a crucial role in enhancing ROS generation and releasing mitochondrial DNA into the cytoplasm, thereby facilitating the activation of NLRP3 inflammasome (17–19). NLRP3 inflammasome acts as a nexus, fostering inflammatory responses and functioning as an effector to initiate the cascade of inflammatory reactions (Figure 1). A critical step in this biological cascade is Caspase-1 activation, an enzyme precursor cleaved into an active form during NLRP3 inflammasome oligomerization (20). Caspase-1 is subsequently responsible for the maturation of pro-Interleukin-1β (pro-IL-1β) and pro-IL-18 into their active form. Gasdermin D (GSDMD)-NT pores formation triggers the processing and secretion of IL-1β and −18. Caspase-1 facilitates pore formation by cleaving various substrates associated with crucial metabolic pathways, actively facilitating the release of IL-1β and IL-18 (21).This culminates in a cascade of inflammatory reactions and the inflammatory infiltration of the myocardium, recognized as pyroptosis (22).

### 2.2 NLRP3 inflammasome as a significant risk factor for myocardial infarction

MI constitutes as a cardiovascular emergency precipitated by arterial thrombosis, resulting in a drastic reduction in myocardial blood flow (23, 24). The pathological features of MI delineate that, signaling pathways involving endothelial cell proliferation, apoptosis, and autophagy, as well as those of fibroblasts, orchestrate myocardial cell injury and the progression of MI. Notably, the NLRP3/Caspase-1-mediated pyroptosis pathway has emerged as a pivotal contributor (25). Research findings (26, 27) highlight that activation of the NLRP3 inflammasome fosters the release of IL-1β and IL-18 from myocardial and arterial endothelial cells, ultimately causing coronary artery impairment and exacerbation of MI. Furthermore, NLRP3 inflammasome activation prompts the release of IL-1β from fibroblasts, thereby instigating fibrotic alterations and promoting the enlargement of infarcted myocardial scars (28). It has been reported that oxidized LDL (oxLDL) within infarcted cardiomyocytes catalyzes cholesterol crystallization and induces the transcription of NLRP3 inflammasome and pro-IL-1β. Subsequent NLRP3 inflammasome activation triggers inflammatory cascade via the Nuclear factor kappa-B (NF-κB) pathway, leading to endothelial cell dysfunction and fostering atherosclerosis (29). Additionally, in accordance with Sandanger et al. (30), upregulation of NLRP3 inflammasome expression alongside pro-inflammatory cytokines IL-1 and IL-18 was documented in MI mice. This affirms, that inflammasome activation in fibroblasts exacerbates myocardial fibrosis and apoptosis, thereby aggravating MI. In summary, NLRP3 emerges as a critical risk factor for MI.

### 2.3 Correlation between NLRP3 inflammasome and congestive heart failure

The initiation of NLRP3 inflammasome resulted in purinergic receptor P2X7 (P2X7R) activation through Adenosine triphosphate (ATP) and diverse toxins. This activation sets off processes like efflux of potassium, the generation of ROS, and mitochondrial DNA release. This cascade results in myocardial damage, myocardial cell necrosis, and the exacerbation of congestive heart failure (CHF) (31). Research indicates that NLRP3 inflammasome activation facilitates IL-1β and −18 extensive release, both of which have the potential to impair myocardial contractility (32). Concurrently, they interfere with the regulation of cytoplasmic calcium by altering the synthesis of proteins, inducing diastolic dysfunction, and subsequently impairing cardiac pumping function, thereby worsening heart failure or potentially inducing heart failure (33, 34). Xin-Li formula (XLF) is a traditional Chinese medicine formulation showing promise in treating heart failure. A study conducted on rats with heart failure induced by combined hyperlipidemia and MI revealed that XLF possesses anti-inflammatory properties. These effects are attributed to its ability to inhibit mammalian target of rapamycin (mTOR) phosphorylation, increasing the population of Foxp3+ T-regulatory cells (Tregs), and inhibiting NLRP3 activation in THP-1 macrophages. This leads to the inhibition of the inflammatory response mediated by NLRP3 inflammasomes, offering potential benefits for patients with heart failure (35). Moreover, Canakinumab is a therapeutic monoclonal antibody targeting IL-1β (36), holds promise in reducing tissue or cell inflammation by blocking IL-1β activity, and potentially inhibiting the inflammatory pathway triggered by the NLRP3 inflammasome. A sub-analysis conducted within the CANTOS trial demonstrated a reduction in the rates of heart failure hospitalization with the administration of canakinumab administration. The MRC-ILA-Heart study (37), which included 182 patients experiencing small acute myocardial infarction (AMI), revealed that NLRP3 inflammasome activation and the heightened IL-1 activity during the acute phase of myocardial infarction correlated with escalating heart failure risk. Canakinumab, conversely, demonstrated the ability to block NLRP3 inflammasome and IL-1β activations, highlighting IL-1-targeted therapy potential in preventing the recurrence of atherosclerotic thrombotic events and heart failure happening (38). In summary, NLRP3 inflammasome activation is implicated in the generation and exacerbation of chronic heart failure (CHF). Intervening within NLRP3 inflammasome pathway may hold promise for improving the prognosis of CHF.

## 3 Correlation between excessive sympathetic nervous system activation and NLRP3 activation

### 3.1 Cardiovascular diseases pathogenesis: dysregulation of the autonomic nervous system

ANS regulation governing cardiac function can be categorized into two systems. Firstly, the central nervous system comprises sympathetic and parasympathetic nerves extending from the brain and spinal cord to the heart. Secondly, the intrinsic cardiac nervous system, constituted by multiple ganglion plexuses (GP) distributed in the epicardial fat pad and within the Marshall ligament, encompassing both sympathetic and parasympathetic nerves. A crucial factor within cardiovascular disease progression is the imbalance in the functions of cardiac sympathetic and parasympathetic nerves. Pathophysiologically, ANS activation, including Sympathetic nervous system (SNS) and renin-angiotensin-aldosterone system (RAAS), serves as determinant within progression and recurrence of cardiovascular diseases (39). Both animal and clinical studies provide substantial evidence indicating that autonomic imbalance in cardiovascular diseases advances from vagal dominance in the early stages to sympathetic dominance in the later stages (40–42). The initial surge in sympathetic activity aims to maintain cardiac output. however, prolonged SNS can lead to increased adrenaline secretion, precipitating changes such as cardiac structural remodeling, deterioration of cardiac function, myocardial cell apoptosis, myocardial tissue fibrosis, and cardiac electrical remodeling. These alterations may manifest as symptoms such as chest tightness, shortness of breath, restricted exercise capacity, or difficulty lying down. Often, these factors can overlap or be causally linked, resulting in a substantial decrease in ejection fraction and severe clinical symptoms and consequences. The unfavorable clinical prognosis observed in patients with cardiovascular diseases is associated with the activation of the SNS, diminished parasympathetic nervous system activity, and reduced heart rate variability (43).

### 3.2 Induction of NLRP3 inflammasome activation by excessive sympathetic nervous system stimulation

**Figure 2:**
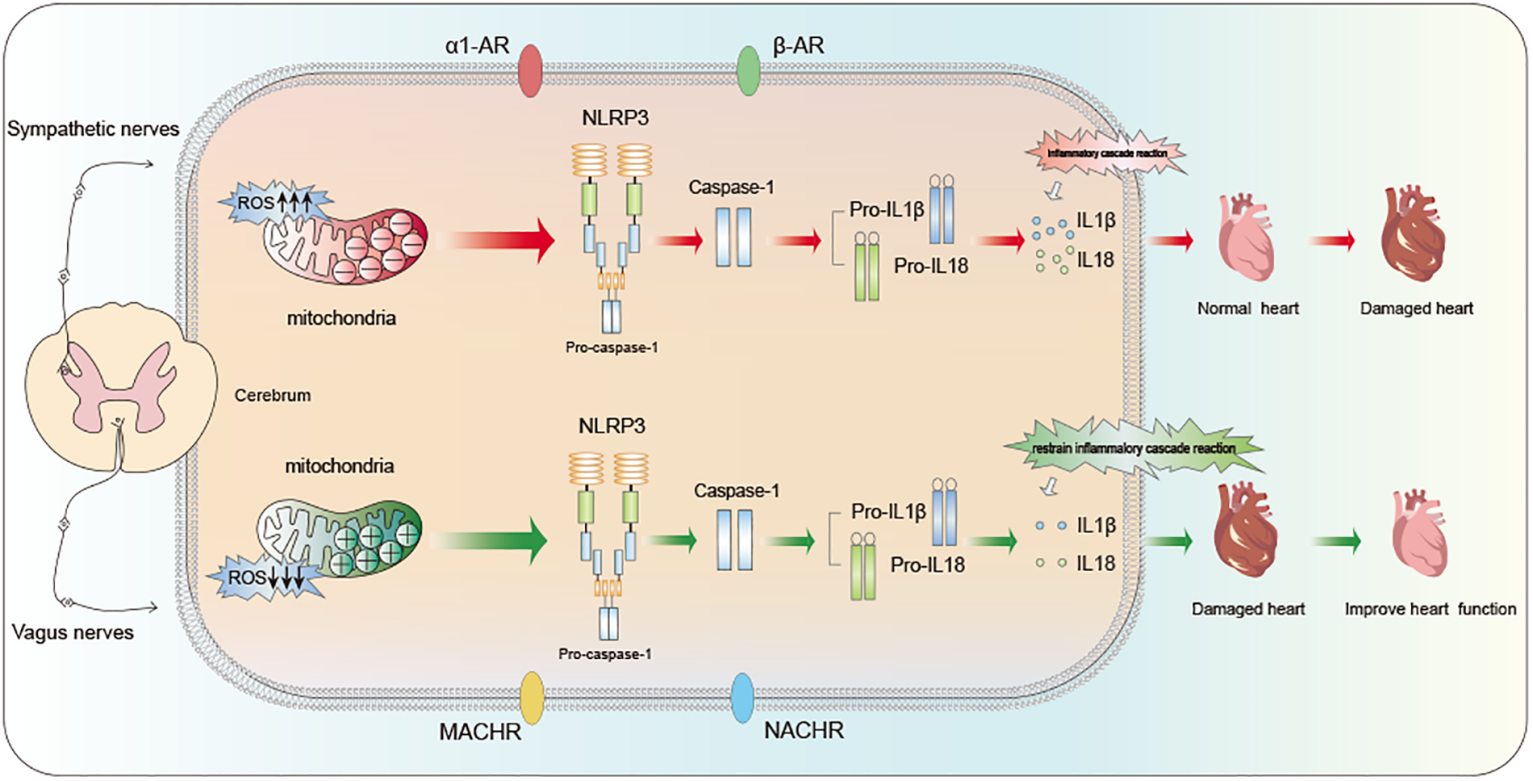
Regulation of cardiac function by ANS: influence mechanism based on NLRP3 inflammasome pathway.

SNS excessive activation is a crucial factor in initiating inflammation and damage to heart. It's stated that the non-selective β-adrenergic receptor agonist isoproterenol (ISO) triggers various pro-inflammatory cytokine expressions within myocardial cells, including tumor necrosis factor (TNF)-α, IL-6, −1β, and −18, as a result of chronic β-adrenergic stimulation (44, 45). These pro-inflammatory cytokines are recognized contributors to myocardial injury and contribute to long-term pathological remodeling, such as myocardial fibrosis. A study on IL-18 in β-adrenergic injury-induced cardiac inflammation and fibrosis validated the findings of the previously mentioned research (46).. This study found that when the heart is excessively stimulated by stress hormones (acute β-adrenergic overstimulation), it triggers certain inflammatory response in heart cell, resulting in harmful changes in the heart structure. This process involves reactive oxygen and receptors called β1-adrenergic receptors. It sets off a chain reaction of cytokines and the infiltration of immune cells (macrophages), ultimately causing negative changes in the heart (Figure 2). SNS has receptors called *α*1-adrenergic receptors (α1-AR). In a significant experiment, using a substance called phenylephrine (PE) that activates these *α*1-adrenergic receptors in mice resulted in heart dysfunction and inflammation (47). Notably, PE injection markedly increased the expression levels of caspase-1, NLRP3, and IL-18 in the heart. In summary, The overstimulation of SNS can trigger the activation of NLRP3, setting off a series of inflammatory responses and ultimately leading to pyroptosis in myocardial cells.

Conversely, additional studies indicated that interrupting β-adrenergic receptor (β-AR) signaling can effectively inhibit IL-1β, NLRP3, and IL-18 activations, subsequently preventing their mediation of cardiac fibrosis (13). Consequently, within cardiovascular disease progression, NLRP3 inflammasome activation induced by excessive sympathetic activation is intricately linked to the progression of cardiovascular diseases. Strategies for inhibiting the overactivation of SNS and promoting VNS to maintain ANS balance present new avenues for therapeutic intervention in cardiac disease treating.

## 4 Mechanisms of VNS within cardiovascular diseases treatment via modulating NLRP3 inflammasome

VNS can effectively inhibit SNS activation by enhancing vagal nerve stimulation, thereby restoring the balance of ANS function. This therapeutic methodology is employed in heart diseases treatment. As previously discussed, excessive SNS activity is closely linked to cardiac inflammation, myocardial injury, NLRP3 inflammasome activation. Recent years have witnessed multiple studies indicating that VNS serves as a determinant in ameliorating cardiac inflammation infiltration, and enhancing ventricular remodeling and improving cardiac function (Figure 2).

### 4.1 Anti-drug induced cardiotoxic effects

Medications, especially cancer drugs, have been known to potentially cause harmful effects on the heart (48). Prolonged administration of drugs may result in myocardial injury and compromised cardiac function, potentially resulting in cardiovascular diseases progression and, in severe cases, progressing to end-stage heart failure. Recent research (49) suggests that VNS therapy may possess cardioprotective properties. It achieves this by alleviating cardiac dysfunction mediated by the NLRP3 inflammasome, enhancing myocardial energy metabolism, reducing inflammatory infiltration, and safeguarding cardiac function in the aftermath of drug-induced injury.

#### 4.1.1 Mitigating doxorubicin-induced cardiotoxicity

Doxorubicin (DOX) serves as one of the common chemotherapy meditation applied for treating various cancers like soft tissue sarcoma, osteosarcoma, breast cancer, bladder cancer, gastric cancer, and thyroid cancer (50). However, a major downside of using DOX is its potential harm to the heart.

In a recent study by Nanthip Prathumsap and colleagues (51), they explored the impact and mechanisms of VNS on DOX-induced cardiotoxicity. The findings confirmed that VNS could confer cardiac protection by enhancing the balance of cardiac sympathetic/parasympathetic nerves, reducing myocardial cell apoptosis, thereby improving left ventricular function, and mitigating cardiac damage. Mechanistically, DOX disrupts cardiac mitochondrial function and dynamics, subsequently activating autophagy in myocardial cells and mitochondria, leading to cardiac inflammation (52). In the course of this process, NLRP3 inflammasome expression and activation increase, promoting pyroptosis within Wistar rats myocardium, which emerges as cell death primary form within Wistar rats myocardial cells processed by DOX (53). Study outcomes indicated that VNS treatment significantly ameliorated DOX-induced mitochondrial and myocardial cell dysfunction, almost entirely abrogating the increase in levels of pyroptosis-related proteins and inflammatory factors induced by NLRP3 inflammasome activation. Additionally, the study revealed that the beneficial effects of VNS could be completely nullified by employing muscarinic acetylcholine receptor (mAChR) or nicotinic acetylcholine receptor (nAChR) inhibitors. In summary, VNS alleviates DOX-induced cardiotoxicity by obstructing NLRP3 inflammasome pathway, playing crucial protective role, potentially mediated through acetylcholine receptors. Although the study suggests that DOX primarily induces myocardial cell death through pyroptosis, additional research is necessary. Techniques like electron microscopy and immunohistochemistry should be employed to clarify the different types of cell death mediated by DOX.

#### 4.1.2 Alleviating trastuzumab-induced cardiotoxicity

Trastuzumab (Trz) is a targeted anticancer medication specifically formulated for the treatment of tumors characterized by human epidermal growth factor receptor 2 (HER2) positivity. While Trz has proven effective in enhancing cancer patient survival, research has substantiated that prolonged use of Trz may result in cardiotoxicity and subsequent cardiac dysfunction (54). Furthermore, extended administration of Trz has been associated with impaired cardiac function, with potential mechanisms of Trastuzumab-induced cardiotoxicity (TIC) encompassing mitochondrial dysfunction, oxidative stress, and inflammation within the heart (55).

In the recent study by Thawatchai Khuanjing et al., they conducted the implantation and stimulation of VNS devices in rats treated with Trz. Additionally, they introduced a treatment group that combined Trz with donepezil (TDPZ), an acetylcholinesterase inhibitor used to mimic VNS functionality (56). The research findings suggest that the hearts of rats treated with Trz exhibit an imbalance between sympathetic and vagal activity, leading to left ventricular dysfunction. Moreover, rats with TIC experience significant damage to mitochondrial function and dynamics, while Trz increases cardiomyocyte death through inducing ferroptosis, apoptosis, and pyroptosis. However, within Trz-treated rats subjected to VNS and TDPZ, mitochondrial ROS levels decrease. The phosphorylation of dynamin-related protein 1 (Drp1), associated with mitochondrial dynamics, and the expression of mitochondrial Drp1 significantly decrease, while fusion protein optic atrophy 1 (OPA1) expression notably increases. These findings affirm that both VNS and donepezil can significantly enhance mitochondrial function and alleviate oxidative stress in cardiomyocytes, offering protection to the heart. Moreover, a crucial factor contributing to cardiomyocyte death is NLRP3 inflammasome-mediated pyroptosis, closely associated with levels of ROS and mitochondrial function (57). Research results indicate that VNS and donepezil similarly alleviate ferroptosis, apoptosis, and pyroptosis in cardiac myocytes.

Collectively, research suggests that VNS or acetylcholinesterase inhibitor application in the context of doxorubicin-induced TIC provides protective effects for the heart. This protection is attributed to the reduction of inflammatory infiltration, improvement in mitochondrial function, suppression of oxidative stress, and inhibition of pyroptosis mediated by NLRP3 inflammasome. Such interventions could potentially be valuable in future treatments for TIC. However, it's important to note that this study simulated VNS effects through acetylcholinesterase inhibitors and did not directly target acetylcholine receptors with specific drugs. VNS therapy is still awaited to be researched.

### 4.2 Cardiac protection against myocardial injury induced by thermal damage

Thermal injury pathogenesis is intricate, involving the confluence of factors such as systemic alterations in cytokine expression, free radical damage, and inadequate tissue perfusion. Thermal injury has the potential to initiate a systemic inflammatory response (SIRS), which may be instigated by the cascade reaction mediated by NLRP3 activation, leading to substantial release of IL-1β (58). Moreover, severe thermal injury has the potential to induce cardiac stress by causing a surge in plasma catecholamines. This leads to stress reactions and heightened sympathetic nerve tension due to increased metabolism, ultimately resulting in myocardial cell damage through the mitochondrial pathway (59). The mitochondrial pathway is a prominent mechanism for myocardial cell death (60). It promotes inflammatory cells infiltration into myocardial tissue by inducing oxidative stress, loss of growth factors, hypoxia, and triggering NLRP3 activation and DNA damage.

In a murine model of thermal injury, Xiaojiong Lu and colleagues delved into the deleterious effects of thermal injury on myocardial tissue and examined VNS impact on myocardial damage. The study underscored the pivotal role of VNS in safeguarding mitochondrial function and suppressing inflammation in myocardial tissue (61). Research findings unveiled notable pathological alterations in mouse myocardial tissue at 6 h and 24 h post-burn injury. At the molecular level, there were observable signs of mitochondrial swelling and dysfunction, coupled by an escalation in ROS linked to oxidative stress and an elevation in NLRP3 inflammasome expression. These observations highlight that thermal injury can lead to significant inflammatory infiltration and damage to the heart, suggesting the potential for post-burn cardiac dysfunction and heart failure (62). Importantly, mice subjected to VNS showed noticeable improvement in myocardial pathology. Critically, VNS effectively reduced thermal injury-induced swelling of myocardial mitochondria and NLRP3 inflammasome expression. Collectively, VNS demonstrates the ability to alleviate myocardial damage caused by thermal injury, potentially by reducing inflammatory infiltration in myocardial tissue induced by NLRP3 inflammasomes. Additionally, the study proposed that VNS might offer protection against cardiac injury through M3-AchR, though further investigation is required.

### 4.3 Application of VNS in myocardial ischemia-reperfusion injury

VNS improves cardiac function by connecting electrodes to the vagus nerve trunk in the neck and a pulse generator (63). In recent years, some studies have advanced VNS devices to enhance therapeutic effectiveness. Sun Yu and colleagues developed a closed-loop self-powered LL-VNS system using triboelectric nanogenerator technology (64). Their results indicated that, even with stimulation intensity much lower than traditional VNS, the system still produced significant therapeutic effects. Moreover, this system significantly reduced inflammatory infiltration of myocardial cells, thus preventing additional damage to the heart cells.

In recent research, Yao Lu and colleagues introduced a magnetic vagus nerve stimulation (mVNS) device. They replaced the traditional vagus nerve electrode with an injectable magnetic hydrogel containing superparamagnetic iron oxide (SPIO) nanoparticles, aiming for more precise and effective vagus nerve stimulation (65). Furthermore, they applied mVNS in an SD rat model and investigated its mechanism of action in myocardial ischemia-reperfusion injury (I/R). The research results revealed that mVNS treatment significantly alleviated myocardial I/R injury and the release of inflammatory cytokines, while inhibiting the expression of OGDHL (a protein associated with increased mitochondrial ROS levels) (66). This reduction in OGDHL expression contributed to less myocardial necrosis caused by myocardial I/R injury. Hence, mVNS could serve as a novel therapeutic approach to inhibit myocardial necrosis, significantly influencing the I/R injury process and improving the prognosis of ischemia-reperfusion myocardium. Additionally, the researchers used the M2AChR antagonist Otenzepad to explore the anti-necrotic mechanism of mVNS. The results showed that Otenzepad attenuated mVNS's function within limiting myocardial infarct size and inhibiting NLRP3 inflammasome activation. Consequently, mVNS may effectively inhibit myocardial necrosis by modulating the M2AChR/OGDHL/ROS axis, protecting the heart, and improving the prognosis of myocardial I/R injury. However, more investigation seems to be necessary to fully delve into the precise mechanism by which acetylcholine receptor activation reduces OGDHL expression.

## 5 Potential therapeutic targets of VNS

Our article has underscored the pivotal role of NLRP3 inflammasome within heart injury and cardiac diseases pathological progression. Moreover, This article elucidates the mechanism by which blocking NLRP3 inflammasome activation via ANS can be utilized to regulate the treatment of heart disease. For instance, studies have unveiled that the excessive activation of β-AR triggers NLRP3 inflammasome in myocardial cells through the β1-AR/ROS signaling pathway, leading to cardiac inflammation and fibrosis. Notably, the blockade of β-AR in C57BL/6 mice significantly alleviates cardiac inflammation, accompanied by a substantial reduction in NLRP3 inflammasome activity (46). Similarly, the inhibition of *α*1-AR in mice treated with PE markedly diminishes heart inflammation induced by NLRP3 inflammasome pathway, resulting in a noticeable improvement in heart function (47).Additionally, VNS exhibits the capacity to curb the overactivation of the SNS and enhance VNS, thereby restoring the balance of ANS function and consequently ameliorating cardiac inflammation and function. Hence, VNS may realize therapeutic objectives in treating heart diseases by diminishing the expression of α1-AR and β-AR in the SNS, thereby inhibiting NLRP3 inflammasome pathway.

The fusion and dysfunction of mitochondria play pivotal roles in the pathological changes associated with heart diseases, resulting in ROS accumulation, where ROS serves as an upstream regulatory factor within NLRP3 inflammasome activation (67). Pertinent researches substantiated that VNS can ameliorate mitochondrial fusion and dysfunction while diminishing ROS production (56, 57, 61). Consequently, mitochondria emerge as a focal point for VNS in cardiac inflammation treatment. Acetylcholine, primary neurotransmitter of the vagus nerve, acts on mAchR or nAchR (68). VNS modulates relevant inflammatory pathways by augmenting acetylcholine release and the expression of mAchR and nAchR. Research indicated that the M2AChR/OGDHL/ROS axis effectively inhibited myocardial cell necrosis (65), affirming the efficacy of this pathway and participation of mAchR. 5 subtypes of muscarinic acetylcholine receptors (M1-5 receptors) have been identified and extensively studied in cardiovascular research. M3AchR is also recognized as a contributor to improve mitochondrial function (69), rendering it a novel and effective potential treating target.

## 6 Conclusion

NLRP3 inflammasome emerges as a pivotal factor triggering cardiac inflammation, exerting unignorable influences on initiation and developing of heart diseases. Dysregulation of the ANS stands out as crucial mediator within NLRP3 inflammasome pathway. This article reviews the roles of SNS activation and VNS in NLRP3 inflammasome and underscores that, despite extensive research on SNS activation and VNS in NLRP3 inflammasome, the precise mechanisms through which VNS regulates NLRP3 inflammasome to ameliorate cardiovascular diseases remain uncertain. In conclusion, the potential of VNS targeting NLRP3 inflammasome shows promise in the treatment of the cardiovascular system. Subsequent research endeavors into the intricate mechanisms governing how VNS regulates NLRP3 inflammasome are imperative for a more comprehensive understanding.
